# Problematising the Discourse of ‘Post-AIDS’

**DOI:** 10.1007/s10912-017-9433-9

**Published:** 2017-02-01

**Authors:** Liz Walker

**Affiliations:** Faculty of Health Sciences, University of Hull, Hull, HU6 7RX United Kingdom

**Keywords:** ‘Post-AIDS’, Normalization, Stigmatization, Chronicity, Global North/South

## Abstract

This paper reflects on the meanings of ‘post-AIDS’ in the Global North and South. I bring together contemporary arguments to suggest that the notion of ‘post-AIDS’ is, at best, misplaced, not least because its starting point remains a biotechnical one. Drawing on aspects of the sub-Saharan African experience, this essay suggests that, despite significant shifts in access to antiretroviral therapy (ART), HIV continues to be fundamentally shaped by economic determinants and social and cultural practices. In this essay, I question the certainty of the discourse of (Western biomedical) ‘positive progress’ (Johnson et al. 2015), which underpins the ‘post-AIDS’ narrative, and suggest that living with HIV and AIDS in our contemporary global context is a life lived with ongoing complexity, stigma and chronicity. I suggest that HIV in the Global North shares many characteristics with HIV in the Global South yet differs in significant ways, not least in the fact that a resource-rich context generates an environment where health and social care support is possible, and, mostly, usual. In both contexts, however, the experience of living with a highly stigmatized illness with no cure in both the Global South and North suggests that this is a point of shared experience.

## Introduction

The language of ‘post-AIDS’ (Persson 2013; Mazanderani and Paparini 2015) is fraught with contradiction – it is a language of seeming possibility – biomedical, political and social. Yet, it is also one which potentially negates the embodied and structural experience of living with HIV and AIDS; the extent to which it remains a ‘disease of signification’ (Treichler 1987); and its social and economic determinants, particularly in the Global South (Auerbach et al. 2011). The political rhetoric of ‘zero new HIV infections, zero discrimination and zero AIDS related deaths’ (UNAIDS 2010) and, more latterly, ‘90 – 90 – 90’ (90% diagnosed; 90% on treatment; 90% virally suppressed, UNAIDS 2014) headlines which talk of ‘an AIDS-free generation’ and ‘ending the AIDS epidemic’ (WHO June 2015) are as eye-catching as they are problematic, no matter the global context in which they are read and engaged, as the everyday experience of living with (and indeed preventing) HIV and/or AIDS is highly complex (Johnston et al. 2015). For many people, it is characterised by uncertainty, anxiety, ongoing negotiation (emotional, social and economic) and the many daily challenges associated with living with an illness without cure. In this paper, I aim to bring together a range of contemporary arguments to suggest that the notion of ‘post-AIDS’ is, at best, misplaced, not least because its starting point remains, primarily, a biotechnical one with anti- retroviral treatment (ART) and a myriad of medical (including male medical circumcision), pharmacological and other technologies at its heart. The discourse, or era, of ‘post-AIDS’, based on the narrative of ‘positive progress’ (Johnson et al. 2015), is not one which can, or should, be embraced unproblematically, for it has the potential to oversimplify a highly complex condition and undermine the centrality of the social determinants and experience of HIV and AIDS. Whilst the ‘post-AIDS’ discourse includes a global public health message and set of targets directed primarily at the Global South, such as an ‘AIDS-free’ generation in Africa, I suggest that this (problematic) discourse also has resonance for the Global North where HIV arguably remains ‘No Ordinary Mainstream Illness’ (Persson et al. 2014). Indeed, living with HIV and AIDS is very ‘present’, and the ongoing experiences of stigma, which are the primary focus of the discussion in this paper, suggest that there is little which is ‘post’ about HIV and AIDS. Historically, the language of HIV and AIDS has always mattered; stigmatising phrases and labels have been profoundly damaging, for example, and the unproblematic use of the language of ‘post-AIDS’ creates the potential to ignore the ongoing social, structural and cultural challenges of HIV and AIDS in both the global South and North. In this essay, I run these contexts side by side, as much to point to the similarities as differences in experience.

## Biotechnical possibilities

There is no doubt that the discourse of ‘post-AIDS’ is made possible because of biomedical innovation and intervention. Antiretroviral drugs have transformed HIV from a life- threatening condition to a manageable, long term, chronic condition (Gilbert and Walker 2010; Whiteside and Strauss 2014; Whiteside 2015). In a biomedical context, at least, ‘HIV is now a very different illness from what it once was even just a decade or two ago’ (Persson et al. 2014, 6). People on appropriate treatment can, and do, live healthy and productive lives. Yin et al. (2014, 4), for example, state that people in the UK living with HIV ‘can expect to live a near-normal life span if they are diagnosed [and treated] promptly’. The same report highlights that one in four people living with HIV is now aged fifty and over. Life expectancy has also risen in low and middle income countries as a result of expanded access to treatment (largely in Africa) although it has not yet returned to pre-1990 levels (UNAIDS 2014). In South Africa, where there are more people living with HIV than any other country, (UNAIDS 2014, 9) people are living on average up to 61.2 years compared with 52.2 years ten years ago (Statistics South Africa 2014). In 2014, ‘life expectancy at birth [was] estimated at 59.1 for males and 63.1 for females’ (SSA 2014).

The shift to living longer with HIV, globally, is due to the increasingly widespread availability and use of anti-retroviral drugs. In the UK, ‘the number and proportion of adults receiving anti-retroviral therapy increased over the past decade. In 2013, 90% (73,300/81,500) of adults seen for HIV care were prescribed ART compared with 69% (28,240/41.160) in 2004. 90% of all adults receiving ART were virally suppressed’ (Yin, et al. 2014, 5). In addition to treatment as prevention (TasP) significantly inhibiting the transmission of HIV (WHO 2015), prevention of infection is also made possible through post-exposure prophylaxis and the ongoing development (and availability) of PrEP (pre-exposure prophylaxis) – which brings with it the joint, yet contradictory, possibilities of control and protection alongside (HIV related) concerns of side effects and managing adherence and stigma (Liu et al. 2014; Young et al. 2014; McCormack et al. 2015). These technical developments in HIV intervention generate their own complexities, requiring an acute and dynamic understanding of the relationships among HIV, medical technologies and the body (Rosengarten 2009).

ART has similarly significantly changed the landscape of HIV and AIDS in sub-Saharan Africa where it is a largely heterosexual epidemic. The estimated overall HIV prevalence rate in South Africa is approximately 11.2% (SSA July 2015). The total number of people living with HIV in South Africa in 2015 is estimated to be approximately 6.19 million. For adults aged 15-49, an estimated 16.59% of the population is HIV positive’ (SSA July 2015). In general terms, access to ART has, without doubt, impacted upon the number of AIDS-related deaths – ‘the decline in the percentage of AIDS-related deaths from 2005 can be attributed to the increase in the roll-out of ART [345 607 in 2005 to 162 445 in 2015]’ (SSA July 2015). However, the same statistical review points to a shift in this trajectory with the recent increase in AIDS deaths between 2014 and 2015 (151 040/162 445), ten years after the roll-out of ART. Despite the now well-rehearsed impact of ART in South Africa, two immediate questions arise from this and other data. The first is the extent of ART coverage and uptake in South Africa (and other sub-Saharan Africa countries), and the second is adherence to medication and the ongoing challenges these pose – both of which point to some of the well-established, but ongoing, limitations of the biomedical approach – limitations and challenges which apply in different social contexts – the richest as well as middle and low income countries (Squire 2010; Mazanderani and Paparini 2015).

In this regard, Whiteside and Strauss (2014, 103) argue that despite the advances in South Africa the most recent data show HIV and AIDS is the largest contributor to the region’s burden of disease’. ART coverage remains uneven throughout southern Africa – in South Africa, it is currently 66% despite having the largest ARV programme in the world and set to expand with the recent decision by the Medicines Control Council (SA) to approve the use of PrEP (MCC, 15 December 2015). Other countries vary: 77% in Zimbabwe; 82% in Zambia; and 50% in Tanzania (Mattes 2014; Whiteside and Strauss 2014). Unpredictable or no access to ART critically impacts on AIDS-related deaths in the short and long term – the cost of treatment remains high and is, of course, long term. As Whiteside and Strauss stress: ‘The HIV epidemic is a long- wave event that needs to be managed long into the future…the timeline for commitment is [thus] far longer than most politicians, strategists and donors are willing to consider, even in their long-term plans’ (2014, 104).

This is underlined by the high number of HIV-infected adolescents in southern Africa (1.2 million) whose long term health is dependent on strict adherence to ART (Hodes and Toska 2015). Yet, ‘adolescent adherence to antiretroviral medication remains lower than adult rates in both high and lower income settings’ (Cluver et al. 2015, 58).

While the provision of ART is lifesaving drug therapy, it is also clearly central to strategies that focus on the prevention of infection. Newly revised WHO guidelines indicate that treatment should be given to all people who are HIV-positive at diagnosis and regardless of CD4 count to reduce onward infection, and to people at risk of contracting HIV (PrEP) (WHO 2015). However, Wilson and Fraser make it very clear that strict adherence to the medication is required to invoke the treatment as prevention effect. Their data indicates that retention of patients on ART in sub-Saharan Africa after three years is only 72% on average (2013). Whiteside and Strauss argue that while the benefits of treatment are clear, TasP can never alone end the AIDS epidemic not only for reasons of retention of people living with HIV on treatment programmes but also because of the issue of viral load, which is highest in newly infected individuals when they are least likely to know that they are infected. A combination of HIV- prevention strategies, including behavioural (for example, condom use) and structural interventions (for example, microfinance schemes aimed at empowering women), they suggest, along with increased access to treatment and care, remains a priority (2014, 107). While these and other data demonstrate the profound, ongoing, life changing impact of biomedical intervention in HIV and AIDS, they also provide both an embodied and discursive space for the articulation of the notion of ‘post AIDS’. The success of ART has reframed the lived experience of HIV undoubtedly but not entirely in the manner suggested by UNAIDS (2014) or indeed, Barack Obama in his most recent reference to the ‘achievement of an AIDS- free generation’ (PEPFAR 28 July 2015). The reasons for this are many and varied and highlight the social, economic and cultural limitations and shortcomings of clinical intervention and technologies (Hunter 2015; Johnson et al. 2015). Nowhere is this more evident than in the experience of HIV and AIDS-related stigma.

## Stigma: the enduring reality

Twenty-five years ago, Susan Sontag ([1989] 1990) wrote of her hope that AIDS would one day become an ‘ordinary illness’. To do this, it would ‘need to be detached from its abundance of unsavoury and dehumanising metaphors, which inflicted untold stigma and elicited so much guilt and shame’ (Persson et al. 2014). This, she predicted, would happen through greater understanding and treatment (Sontag [1989] 1990). Whilst there is no doubt that developments in treatment and concomitant attitudes towards people who have a diagnosis of HIV have altered the personal, social and political status of the condition, Sontag’s vision has yet to be fully realised. In the context of the discussion here, it is, perhaps, in the Global South that this disparity is most acutely felt. Indeed, ‘positive progress narratives on HIV’ should be used with the most exquisite sensitivity (if not reluctance), as many working in southern Africa, in particular, acknowledge, both publicly and politically, that the lived experience (and daily reality) of HIV/AIDS is very far from the rhetoric which is reflected in the discourse of ‘post-AIDS’. The language of international health policy is, in this context, significantly at odds with those infected and affected by HIV and AIDS. Arguably, it is the Western neoliberal political imperative to ‘conquer the AIDS epidemic’, to ‘save Africa (in particular), from itself’ and to ‘celebrate scientific achievement’, which is embedded in the ‘post-AIDS’ narrative – a ‘progress’ narrative which seeks to assert control in a context of global conflict, insecurity and uncertainty (Booth 2010; Ingram 2011).

One consequence of the biomedical treatment prerogative in the context of HIV and the critical issue which underpins the notion of a ‘post-AIDS’ era has been the drive to normalise what is ‘one of the most infectious diseases in history’ (Persson 2013,1065). Normalisation, wherein HIV undergoes a semantic transformation from ‘death sentence’ to ‘chronic illness’, is the critical driver for new approaches and attitudes towards HIV. As such, with ‘the introduction of new treatments in the mid-1990s and their dramatic effects on mortality and morbidity, along with political activism and the realisation that the virus is not limited to particular populations, nor anywhere near as infectious as first feared, HIV has become repositioned in Western public discourse as a manageable chronic illness’ (Persson 2013, 1067; Owen and Catalan 2012). Indeed, as Squire suggests, if HIV becomes normalised, people living with HIV, ‘turn into regular, unremarkable citizens, just like anyone else’, becoming ‘doubly normalised’, both as having a familiar health condition and one which is comparable with other long-term conditions (2010, 408). However, as Beuthin et al. suggest in their work in Canada on ageing with HIV, ‘this view is misleading and requires caution’ (2014, 612). The seemingly innocuous linguistic shift (and one used with growing regularity), however, whilst representative of *some* profound changes in the ways in which HIV is perceived, legislated for, lived and understood, has not, I would argue, actually shifted the lived experience of HIV to such an extent as to allow for a reading of the infected person as stigma free. It is, therefore, ‘premature to argue that HIV is now widely perceived in developed countries as a mainstream chronic disease on par with diabetes and asthma, or that HIV does not carry some continuing and residual stigma in the community, including in parts of the health care sector’ (Persson et al. 2013, 6).

Indeed, research on HIV-related stigma suggests that, in contrast to Sontag’s ([1989] 1990) early hopes, the embodied experience of being HIV positive can remain one of conflict, derision, ambiguity, and, often, distress. Despite the drive to normalise HIV and to transform it into a disease ‘like any other’ (Moyer and Hardon 2014, 263; Mazanderani and Paparini 2015, 66; Murphy et al. 2015), people continue to live in what might now be termed a liminal illness space wherein normalcy and exceptionality are uncomfortable, yet ever present, bedfellows – it is, thus, simultaneously normal and exceptional (Mazanderani and Paparini 2015), which makes for a tension filled experience for people diagnosed, as they are expected to embody and perform the ‘normal (chronic illness)’ whilst, perhaps, often experiencing their own condition as entirely exceptional. As such, a further pressure is also brought to bear, as those people who fail to achieve normalcy in either their understanding or performance of HIV risk a new and unanticipated form of stigma which is grounded in the increasingly demanding biomedical prerogative and expectation that someone with HIV will respond ‘appropriately’ to treatment both psychologically and physically. That is, they ‘should’ not internalise the still very evident stigma surrounding HIV, they will adhere to treatment protocols and, most importantly, they will not die from the condition. Indeed, to do so would profoundly undermine contemporary medical and social expectations.

Furthermore, this contemporary discourse potentially undermines the support (aside from the biomedical) that those diagnosed with HIV might have previously relied upon. For, if a condition is ‘normal, ‘everyday’ and ‘unexceptional’, then the need for ‘exceptional’ support is no longer present. There is, thus, a clear political imperative at work here in both the Global North and South where it manifests differently, which allows for, with disastrous consequences, a potential withdrawing of social, political and, perhaps most importantly, financial assistance and support (Squire 2010; Mazanderani and Paparini 2015). Perhaps the impact of the drive to ‘becoming ordinary’ and to ‘normalcy’ is that ‘the discursive normalisation of HIV as a disease like any other’ (Mazanderani and Paparini 2015, 66) may ironically constitute a further mechanism to generate silence, fear and a continuation of old, and generation of quite new, forms of HIV related stigma.

HIV remains socially constructed, therefore, in ways that biomedicine cannot and has not altered. Clinical intervention has transformed clinical outcomes, but the social, economic, and gendered determinants of HIV remain largely intact, as is evident through its global epidemiological profile. In the South African context, for example, the relationship between poverty, socioeconomic status, socio-cultural norms, gender and HIV risk are highly complex and made more so by very high levels of gender-based violence and serious inadequacies in the health services which impact on the diagnosis and management of HIV, AIDS and TB (Natrass et al. 2012; Natrass 2014; Hunter 2015; Johnston et al. 2015). Navigating and managing relationships and safe sexual practices, including HIV testing and condom use, in this context is understandably fraught, where the influences of economic insecurity, unemployment, socio-economic inequality and long term historical patterns of migration will often have the last, if not determining, word (Hunter 2007; Hosegood 2009; Mindry et al. 2012). The HIV epidemic in South Africa thus continues to impact negatively and unevenly on individuals, families and communities despite the clear and evident advances in limiting and managing the epidemic through a range of interventions including ART. HIV and AIDS intersects with, and exacerbates, existing social and health inequalities. In the context of the Global South, in particular, then, it is unsurprising that HIV- related stigma continues to be a major challenge, despite the growing presence and roll out of effective treatment. Indeed, a growing body of scholarship in recent years has sought to explore the enduring impact of ART in relation to ageing with HIV and the interrelationship between the long-term effects of medication and the development of particular co-morbidities. This work has also drawn attention to psychosocial changes and stressors, associated with long-term side effects of ART – the enduring, if shifting, impact of stigma remains central to living with HIV (Owen and Catalan 2012; Meyers and Lawrence 2013; Squire 2013; Beuthin et al. 2014).

## Living with HIV and AIDS-related stigma

The recent ‘People Living With HIV Stigma Index: South Africa 2014’ study (May 2015) was conducted with 10,473 people, all of whom were HIV positive. It concluded that there is still a ‘moderate’ level of stigma which affected one third of the population of people living with HIV who took part in the study. According to the report, it was, in fact, internalised stigma which posed the greatest challenge with 43% of the respondents expressing feelings of internalised stigma: of these, 29% felt ashamed, 28% had feelings of guilt, 31% blamed themselves, 19% blamed others and 22% had low self-esteem. Interestingly, the respondents reported that experiences of internalised stigma, in the twelve months prior to reporting, were more frequent than those that were externally mediated. 11% of respondents felt that they should be punished because of their HIV status, whilst 11% also reported feeling suicidal. The result of these feelings and experiences was that people engaged in social avoidance and actively changed their behaviours (not getting married, not having children, etc.) in this context. Whiteside points to the rise in South Africa’s maternal mortality rate (from 230 deaths per 100,000 live births in 1990 to 410 in 2008) – an indicator which he suggests is a direct consequence of social stigma yet is not recognised and treated as such (2015, 463). These studies indicate that, while explicit discrimination on the basis of HIV has altered, feelings of shame, blame and the need to adapt life choices to accommodate mental and physical distress are very much part of the everyday lived experience of HIV in South Africa.

Other research in SSA demonstrates similar trends. One study, for example, which explored ‘life time journeys on antiretroviral drugs’, by Mbonye et al. (2013) demonstrates the varying, yet ongoing, impact of HIV-related stigma. In this study, undertaken in Uganda, involving interviews with people on ART over a thirty month period – at three, six, eighteen and thirty months, Mbonye et al. show that there were significant changes after the introduction of ART which did help to reduce stigma. However, as people living with HIV worked to rebuild their lives, the long term (bodily) side effects of ART began to surface, as did a familiar negative response to their reintegration into their social and economic networks. Recovery, through ART, in some ways required the construction of an ‘HIV-free’/‘HIV-well’ identity which their respondents found was challenging to maintain. The researchers observed that, ‘for some participants, ART was no longer the magic bullet that was taking care of the stigmatising signs on their bodies. In fact, these people had started connecting the new forms of strange bodily signs with ART, which had the potential to bring back strong feelings of shame (6). For example, one of their respondents interviewed after thirty months on ART said,

You see my mouth has changed its shape and even my face is pale with sunken eyes. Even though I try to add some Vaseline on my face it doesn’t change. People can think that I don’t bathe properly according to the way my skin looks. As a human being, I feel bad but there is nothing to do about it. I don’t mind if one talks about me and I can’t quarrel with him or her but in my mind it hurts me. (6).

These findings are echoed by Mburu et al. who also suggest that, in contrast to suggestions that stigma may not be relevant in the context of a mature HIV epidemic, ‘stigma remains a concern among people living with HIV in Uganda, where antiretroviral coverage is estimated to be between 52 and 81%’ (2013,5). Like the South African Stigma Index (2015), Mbonye et al.'s (2013) study also indicated that respondents made a number of behavioural changes (stigma avoidance strategies) in order to ameliorate and accommodate the effects of HIV-related stigma. The process of ‘sero –sorting’ was one such strategy whereby people seek relationships with people who are also HIV positive to offset the need to respond to customary questions around HIV status. One respondent summarised this:

Let me tell you when there is health, there is life. I tried very much to be single but when I couldn’t hold on any longer I got someone who was also with TASO [The AIDS Support Organisation] and is on medication. (Mbonye et al. 2013, 8).

Another strategy identified in the research included accessing treatment from different geographic locations. Gilbert and Walker (2009a, b), in a study of patient experiences of ART, in Johannesburg, South Africa, found that taking ART, whilst life-saving, rendered their HIV positive status both visible (through clinic attendance and taking pills) and stigmatising. As a result, many respondents in the study suggested, or admitted, for example, that they chose not to use clinics in their local areas in order to avoid this form of stigmatisation from members of their families and the wider community. One young woman, for example, said:

The community is not very supportive. They speak very negatively about HIV infected people, hence I travel from Port Elizabeth to Johannesburg (1200km) to receive my medication to spare my mother the shame in the community, as she is a very well-known person in the community. (2009a, 143).

One of the central findings of this research was that the intensity of HIV- related stigma threatened to compromise the value of ART: for some people, the fear of the consequences of disclosure were greater than the prospect of feeling better due to ART.

Further studies in sub-Saharan Africa demonstrate similar findings. Maughan-Brown (2010), for example, demonstrates the tenacity of stigma despite ART, whilst Parsons et al. (2015), in a study with people with disabilities who are HIV positive in Zambia, focussed on the ways in which intersectional vulnerabilities are further underlined and exacerbated by a diagnosis of HIV. They concluded that:

…the experiences outlined by our participants indicate that stigma importantly affects the ways in which PWD/HIV+ are able to manage their health conditions. As we and other researchers have shown, stigmatised individuals are more likely to feel ashamed, hide their HIV status, and discontinue ART, which may not only result in tragic consequences for the individual, but a great likelihood of transmission of HIV into the broader community (for drug-resistant strains). (Parsons et at. 2015, 17)

What all these studies in southern and sub-Saharan Africa demonstrate is that stigma in its various forms continues in the face of ART. Moreover, recent biomedical advances in the treatment of HIV seem potentially compromised by the tenacity of HIV-related stigma and its tendency to morph into perhaps less overt, but perpetually insidious, and damaging forms.

In the context of the Global North, of course, there are parallels and critical divergences in people’s experiences of ongoing stigma in the context of ART and the ways in which the ‘post-AIDS’ and ‘normalisation’ rhetoric might not take sufficient cognisance of the ongoing experiences of living with HIV, of which stigma remains one (Dodds 2006; Flowers et al. 2011; Meyers and Lawrence 2013; Murphy et al. 2015). A number of surveys carried out in the UK over the past five years provide evidence of ongoing concerns and anxieties about discrimination, disclosure and stigma (National AIDS Trust 2010, 2011; Positively UK 2013). Yin et al., for example, report positive health and health care experiences despite ‘a high prevalence of co-morbidities, mental health issues, and experiences with stigma and discrimination among respondents’ (2014, 25). Chinouya et al., based on findings from the UK Stigma Index Study, highlight the nature of stigma experienced by migrants living with HIV in the UK (2014, 4). Of significance here is the finding that ‘felt stigma was reported as salient. Fifty-nine percent of men and 48% of women indicated that they felt guilty living with HIV’. In much the same way as research participants in Ugandan and other studies highlighted earlier, participants in this study also reported that the shame of living with HIV resulted in avoiding clinic appointments and decisions to avoid intimate relationships (Chinouya et al. 2014, 7). The tenacity of HIV-related stigma is striking in all of these contexts and none more so than in the narrative case study presented by Beuthin et al. in the context of ageing with HIV in Canada where long term use of ART is normalised. Having lived with HIV for more than twenty years, Nancy says,

…the stigma of HIV is still so great. You live with fear that you might pass it on. And having HIV is not like having diabetes. You cannot date, and now it has this criminal element and it is stigma personified…. It’s an ugly problem and I feel humiliation… The stigma is always there, the threat of it. It’s like wearing a back pack. I’m never sure when I can take it off and set it down on safe ground’ (2015, 615).

## What does it mean to be HIV positive in the era of ‘post-AIDS’?

In the context of HIV and AIDS, language has always been a crucial tool used to generate and perpetuate attitudes and approaches. In the past, the ‘gay plague’, ‘GRID’, the ‘African disease’ and other such terms have been centrally implicated in the development of HIV-related stigma. In short, the language of AIDS matters and, in this context, the language of ‘post-AIDS’, in particular, has its own semantic and linguistic resonance. It is a term which is finding prominence (politically and discursively), perhaps uncritically, with significant implications for what it is to be an ‘HIV citizen’: to be a citizen with HIV in the ‘post-AIDS’ era, means, amongst many other things, embodying a quite new HIV identity. In many ways, it is one in which positivity and active resistance situates the ‘new’ ‘normalised’ HIV identity in direct opposition to traditional historical tropes of inevitable decline and death. ‘Post-AIDS’ is premised not just on the political and economic commitment to treatment and prevention but, fundamentally, on the ‘normalisation’ of HIV. Yet, the preceding discussion on stigma clearly suggests that, despite the extraordinary global impact of ART, HIV and AIDS are not ‘normal’ or like any other ‘ordinary’ illnesses. Indeed, as Squire argues, HIV cannot be rendered normal for three reasons (2010, 409). The first is because of ‘its associations with pathologised social states – sex work, intravenous drug use and gay, female and ‘promiscuous’ sexualities – and its potentially fatal, difficult to treat nature’. The second, she suggests, is because ART does not normalise it medically – the long term consequences of treatment and their stigmatising bodily effects unsettles ‘treatment optimism’ and the possibilities held by TasP, and, finally, people living with HIV are not in a position to ‘relate ‘normally’….to work, parenting and relationships’ [and]……. Nor can HIV-negative or unknown status people always conduct themselves in a normalised way in relation to HIV, treating it without stigmatisation…’ (410). The pursuit of normalisation has always been at the heart of the enduring struggle to oppose HIV-related stigma, yet living with HIV and AIDS in countries with both high and low standards of health care remains ‘exceptional’ yet ‘normalised’, contradictory but mainstream. The everyday lived experience of HIV and AIDS where many people may move/tumble between the two, and of living with chronicity if you are fortunate enough to have consistent access to treatment, is fraught with challenges and victories. It is an illness journey that is complex, socially and culturally formed, characterised by the existence of many co-morbidities, and continues to remain more than ‘just a virus’. The danger of the language and practice of ‘post-AIDS’ and the rhetoric of ‘zero discrimination’ is that the voice of complexity is not heard; the voice of long-term chronicity may be silenced, and those who may be unable to articulate their account of HIV and AIDS because of social and health inequalities run the risk of being ignored in the era of ‘post-AIDS’ (Mazanderani and Paparini 2015). The extraordinary efficacy of ART has both made possible, and ironically eclipsed, everyday life with HIV and AIDS; yet, we need to be cautious of taking for granted, or embracing, a new era, underpinned by an uncomplicated and uncritical language of normalisation which may ironically undermine the very efficacy which facilitated the discourse of ‘post-AIDS’ in the first place. What it means to live with HIV and AIDS in our contemporary global context remains as fundamental a question now as it did thirty years ago. It is a life lived with ongoing complexity, stigma and chronicity despite and, as I have outlined, perhaps also because of, its changing biotechnical context.

## References

[CR1] Auerbach JD, Parkhurst J, Caceres C (2011). Addressing the Social Drivers of HIV/AIDS for the long-term Response: Conceptual and Methodological Considerations. Global Public Health.

[CR2] Beuthin RE, Bruce A, Sheilds L (2014). Storylines of Aging with HIV: Shifts towards Sense Making. Qualitative Health Research.

[CR3] Booth K (2010). A Magic Bullet for the ‘African Mother’? Neo-imperial Reproductive Futureism and the Pharmaceutical ‘Solution’ to the HIV/AIDS Crisis. Social Politics.

[CR4] Chinouya M, Hildreth A, Goodall D, Aspinall P, Hudson A (2014). Migrants and HIV Stigma: Findings from the Stigma Index Study (UK). Health and Social Care in the Community.

[CR5] Cluver L, Hodes RJ, Toska E, Kidia KK, Mark Orkin F, Sherr L, Meinck F (2015). ‘HIV is like a Tsotsi. ARVs are your Guns’: Associations between HIV-disclosure and Adherence to Antiretroviral Treatment among Adolescents in South Africa. AIDS.

[CR6] Department of Health, South Africa. 2015. *Medicines Control Council approves Fixed-dose Combination of Tenofovir Disproxyl Fumarate and Emtricitabine for Rre-exposure Prophylaxis of HIV.* Accessed 21 January 2016. http://www.mccza.com/documents/2e4b3a5310.11_Media_release_ARV_FDC_PrEP_Nov15_v1.pdf.

[CR7] Dodds C (2006). HIV-related Stigma in England: Experiences of Gay Men and Heterosexual African Migrants Living with HIV. Journal of Community and Applied Social Psychology.

[CR8] Flowers, Paul, Mark McGregor Davis, Michael Larkin, Stephanie Church and Claire Marriott. 2011. “Understanding the Impact of HIV Diagnosis among Gay Men in Scotland: An Interpretive Phenomenological Analysis.” *Psychology and Health* 26: 1378-1391. 10.1080/08870446.2010.551213 22010635

[CR9] Gilbert L, Walker L (2009). They (ARVs) are my life, without them I am nothing’ – Experiences of Patients attending a HIV/AIDS Clinic in Johannesburg, South Africa. Health and Place.

[CR10] -----. 2009b. “‘My biggest fear was that people would reject me once they knew my status…’ – Stigma as Experiences by Patients in an HIV/AIDS Clinic in Johannesburg, South Africa.” *Health and Social Care in the Community* 18:139-146. 10.1111/j.1365-2524.2009.00881.x 19708868

[CR11] Hodes, Rebecca and Elona Toska. 2015. “Sex and Secrecy: Knowledge of HIV Status, Disclosure and Sexual Practices among HIV-positive Adolescents in South Africa.” Centre for Social Science Research, University of Cape Town, 24 March. Accessed 19 January 2017. http://www.cssr.uct.ac.za/events/2015/sex-and-secrecy-knowledge- hiv-status-disclosure-and-sexual-practices.

[CR12] Hosegood V (2009). Demographic Impact of HIV and AIDS on the Family and Household Life-cycle: Implications for Strengthening Families. AIDS Care.

[CR13] Hunter M (2007). The Changing Political Economy of Sex in South Africa: The Significance of Unemployment and Inequalities to the Scale of the AIDS Pandemic. Social Science and Medicine.

[CR14] Hunter Mark (2015). The political economy of concurrent partners: toward a history of sex–love–gift connections in the time of AIDS. Review of African Political Economy.

[CR15] Ingram A (2011). The Pentagon’s HIV/AIDS Programmes: Governmentality, Political Economy, Security. Geopolitics.

[CR16] Johnston D, Deane K, Rizzo M (2015). The Political Economy of HIV. Review of African Political Economy.

[CR17] Liu A, Cohen S, Follansbee S, Cohen D, Webber S, Sachdev D, Buchbinder S (2014). Early Experiences Implementing Pre-exposure Prohylaxis (PrEP) for HIV Prevention in San Francisco. PLOS Medicine.

[CR18] Maughan-Brown B (2010). Stigma Rises despite ARV Roll Out – A Longitudinal Analysis in South Africa. Social Science and Medicine.

[CR19] Mattes D (2014). Caught in Transition: The Struggle to Live a ‘Normal’ Life with HIV in Tanzania. Medical Anthropology.

[CR20] Mazanderani F, Paparini S (2015). The Stories We Tell: Qualitative Research Interviews, Talking Technologies and the ‘normalisation’ of Life with HIV. Social Science and Medicine.

[CR21] Mbonye M, Nakamanya S, Birungi J, King R, Seeley J, Jaffar S (2013). Stigma Trajectories among People Living with HIC (PLHIV) Embarking on a Life Time Journey with Antiretroviral Drugs in Jinja, Uganda. BMC Public Health.

[CR22] Mburu, Gitua, Mala Ram, Morten Skodval, David Bitira, Ian Hodgson, Grace Mwai, Chistine Stegling and Janet Seeley. 2013. “Resisting and challenging stigma in Uganda: the Role of Support Groups of People living with HIV.” *Journal of the International AIDS Society*. doi.org/10.7448/IAS.16.3.18636. 10.7448/IAS.16.3.18636 PMC3833188 24242256

[CR23] McCormack S, Dunn DT, Desai M, Dolling DI, Gafos M, Gilson R, Sullivan AK, Clake A, Svhembri G, Mackie N, Bowman C, Lacey CJ, Apea V, Brady M, Fox J, Taylor S, Antonucci S, Khoo SH, Rooney J, Nardone A, Fischer M, McOwan A, Phillips AN, Johnson AM, Gazzard B, Gill. ON (2015). Pre-exposure Prophylaxis to Prevent the Acquisition of HIV-1 Infection (PROUD): Effectiveness Results from the Pilot Phase of a Pragmatic Open-label Randomised Trial. The Lancet.

[CR24] Meyers S, Lawrence J (2013). What do Gay Men living with HIV Want for Their Older Age? The Results of a Collaborative Research Project in the North West of England.

[CR25] Mindry D, Maman S, Chirowodza A, Muravha T, van Royen H, Coates T (2012). Looking to the Future: South African Men and Women Negotiating HIV risk and Relationship Intimacy. Culture, Health and Sexuality.

[CR26] Moyer E, Hardon A (2014). A Disease Unlike Any Other? Why HIV Remains Exceptional in the Age of Treatment. Medical Anthropology.

[CR27] Murphy, Patrick. J, David Hevey, Siobann O’Dea, Neans Ni Rathaille and Fiona Mulcahy. 2015. “Serostatus Disclosure, Stigma Resistance, and Identity Management among HIV-Positive Gay Men in Ireland." *Qualitative Health Research*. doi:1049732315606687. 10.1177/1049732315606687 26386024

[CR28] National AIDS Trust (2010). HIV and Black Caribbean Communities in the UK.

[CR29] National AIDS Trust (2011). The Impact of Social Care Support for People Living with HIV. The results of NATs Snapshot Survey of Healthcare Professionals.

[CR30] Natrass N (2014). Millennium Development Goal 6: AIDS and the International Health Agenda. Journal of Human Development and Capabilities.

[CR31] Natrass N, Maughan-Brown B, Seekings J, Whiteside A (2012). Poverty, Sexual Behavior, Gender and HIV Infection among Young Black Men and Women in Cape Town, South Africa. African Journal of AIDS Research.

[CR32] Owen G, Catalan J (2012). ‘We never expected this to happen’: Narratives of Ageing with HIV among Gay Men Living in London, UK. Culture, Health and Sexuality.

[CR33] Parsons J, Bond VA, Nixon S (2015). ‘Are we not human?’ Stories of Stigma, Disability and HIV from Lusaka, Zambia and their Implications for Access to Health Services. PLOS ONE.

[CR34] Persson A (2013). Non/infectious Corporealities: Tensions in the Biomedical Era of ‘HIV Nnormalization.’. Sociology of Health and Illness.

[CR35] Persson A, Newman C, Hopwood M, Kidd MR, Canavan PG, Kippax SC, Reynolds RH, De Wit JBF (2014). No Ordinary Mainstream Illness: How HIV Doctors Perceive the Virus. Qualitative Health Research.

[CR36] Positively UK (2013). States of Mind: Improving Mental Wellbeing in the HIV Community.

[CR37] Rosengarten M (2009). HIV Interventions. Biomedicine and the Traffic between Information and Flesh.

[CR38] Treichler P (1987). AIDS, Homophobia, and Biomedical Discourse: An Epidemic of Signification. Cultural Studies.

[CR39] Sontag, Susan. (1989) 1990. *Illness as metaphor and AIDS and Its Metaphors*. London: Penguin Books.

[CR40] South African National AIDS Council (2015). The People Living With HIV Stigma Index: South Africa 2014 Summary Report.

[CR41] Statistics South Africa (2014). Stats in Brief.

[CR42] Squire C (2010). Being Naturalised, Being Left Behind: the HIV Citizen in the Era of Treatment Possibility. Critical Public Health.

[CR43] Squire C (2013). Living with HIV and ARVs: Three-Letter Lives.

[CR44] UNAIDS. 2010. *UNAIDS Strategy 2011-2015 Getting to Zero*. Accessed 8 November 2015. http://files.unaids.org/en/media/unaids/contentassets/documents/unaidspublication/2010/JC2034_UNAIDS_Strategy_en.pdf.

[CR45] UNAIDS. 2014. *90-90-90: An Ambitious Treatment Target to Help End the AIDS epidemic.* Accessed 8 November 2015. http://www.unaids.org/en/resources/documents/2014/90-90-90.

[CR46] United States President’s Emergency Plan for AIDS Relief. 2015. *President Barack Obama’s Historic Trip to Kenya & Ethopia*. http://www.pepfar.gov/press/releases/2015/245401.htm.

[CR47] Whiteside A (2015). The Key Question in the AIDS Epidemic in 2015. Review of African Political Economy.

[CR48] Whiteside A, Strauss M (2014). The End of AIDS: Possibility or Pipe Dream? A Tale of Transitions. African Journal of AIDS Research.

[CR49] Wilson D, Fraser N (2013). Who Pays and Why? Costs, Effectiveness, and Feasibility of HIV Treatment as Prevention. Clinical Infectious Diseases.

[CR50] World Health Organisation. 2015. Guideline on When to Start Antiretroviral Therapy and on Pre-exposure Prophylaxis for HIV.” http://www.who.int/hiv/pub/guidelines/earlyrelease-arv/en/. 26598776

[CR51] Yin, Zheng, Alison Brown, Gwenda Hughes, Anthony Nardone, O.Noel Gill and Valerie Delpech. 2014. *HIV in the United Kingdom 2014 Report.* https://www.gov.uk/government/uploads/system/uploads/attachment_data/file/401662/2014_PHE_HIV_annual_report_draft_Final_07-01-2015.pdf.

[CR52] Young I, Flowers P, McDaid L (2014). Barriers to Uptake and Use of Pre-exposure Prophylaxis (PrEP) among Communities most Affected by HIV in the UK: Findings from a Qualitative Study in Scotland. BMJ Open.

